# Evidence of dark oxygen production at the abyssal seafloor

**DOI:** 10.1038/s41561-024-01480-8

**Published:** 2024-07-22

**Authors:** Andrew K. Sweetman, Alycia J. Smith, Danielle S. W. de Jonge, Tobias Hahn, Peter Schroedl, Michael Silverstein, Claire Andrade, R. Lawrence Edwards, Alastair J. M. Lough, Clare Woulds, William B. Homoky, Andrea Koschinsky, Sebastian Fuchs, Thomas Kuhn, Franz Geiger, Jeffrey J. Marlow

**Affiliations:** 1 The Scottish Association for Marine Science, (SAMS), Oban, UK; 2 Heriot-Watt University, Edinburgh, UK; 3 GEOMAR Helmholtz Centre for Ocean Research Kiel, Kiel, Germany; 4 Department of Biology, Boston University, Boston, MA USA; 5 Bioinformatics Program, Boston University, Boston, MA USA; 6 Department of Earth and Environmental Science, University of Minnesota, Minneapolis, MN USA; 7 Faculty of Environment, University of Leeds, Leeds, UK; 8 School of Science, Physics and Earth Sciences, Constructor University Bremen, Bremen, Germany; 9 Federal Institute for Geoscience and Natural Resources (BGR), Hannover, Germany; 10 Technological Institute, Northwestern University, Evanston, IL USA

**Keywords:** Marine biology, Marine chemistry, Environmental chemistry, Environmental impact, Element cycles

## Abstract

Deep-seafloor organisms consume oxygen, which can be measured by in situ benthic chamber experiments. Here we report such experiments at the polymetallic nodule-covered abyssal seafloor in the Pacific Ocean in which oxygen increased over two days to more than three times the background concentration, which from ex situ incubations we attribute to the polymetallic nodules. Given high voltage potentials (up to 0.95 V) on nodule surfaces, we hypothesize that seawater electrolysis may contribute to this dark oxygen production.

## Abstract (web summary)

Oxygen is generated abiotically at the abyssal seafloor in the presence of polymetallic nodules, potentially by seawater electrolysis, according to in situ chamber and ex situ incubation experiments.

## Main

**Extended Data Fig. 1:**
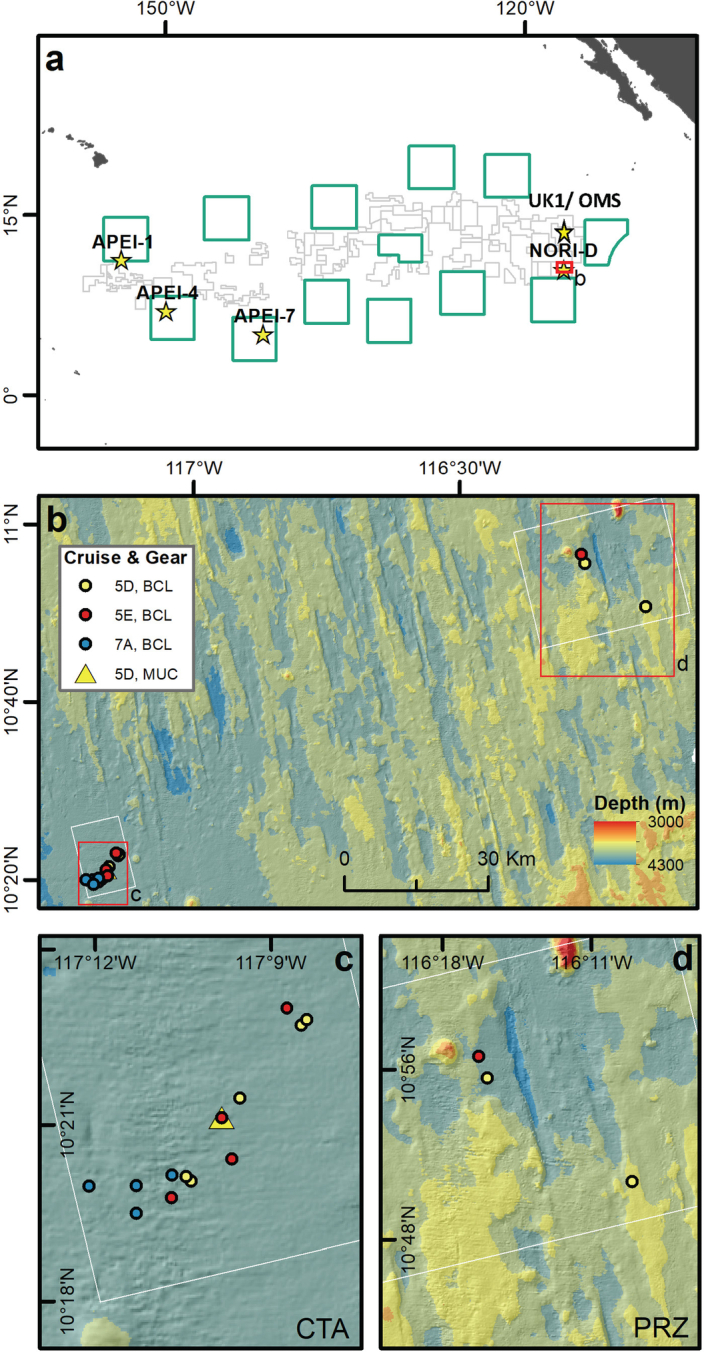
Benthic chamber lander and multi-corer deployment locations across the CCZ. Benthic chamber lander (BCL) locations in APEIs 1, 4, and 7 (western CCZ), UK1 and OMS and NORI-D (stars) (a) and both areas (Collector Test Area or CTA and Preservation Reference Zone or PRZ) (b–d) of NORI-D in the central abyssal Pacific. The deployment location for the multi-corer (MUC) that sampled sediments for the ex situ experiments conducted during the 5D cruise is also shown (c).

Oxygen (O_2_) is prevalent in deep-sea surface sediments where its rate of consumption reflects the sum of aerobic respiration and oxidation of reduced inorganic compounds produced by anaerobic decay. These processes define sediment community O_2_ consumption (SCOC), and quantifying SCOC is needed to estimate fluxes of major elemental cycles through marine systems^1–3^. We undertook multiple in situ benthic chamber lander experiments to measure abyssal SCOC in the Nauru Ocean Resources Inc. (NORI)-D licence area of the Clarion–Clipperton Zone (CCZ; Extended Data Fig. 1 and Extended Data Table 1) where polymetallic nodules cover extensive areas of seafloor. Sediments and nodules were exposed to different experimental treatments, which included the addition of dead-algal biomass, dissolved inorganic carbon and ammonium (NH_4_^+^) or cold filtered surface seawater. No-injection controls were also performed. In contrast to previous deep-sea O_2_ flux studies that only showed SCOC, we consistently found that more O_2_ was accumulating in the chambers than was being consumed, resulting in net O_2_ production.

**Fig. 1:**
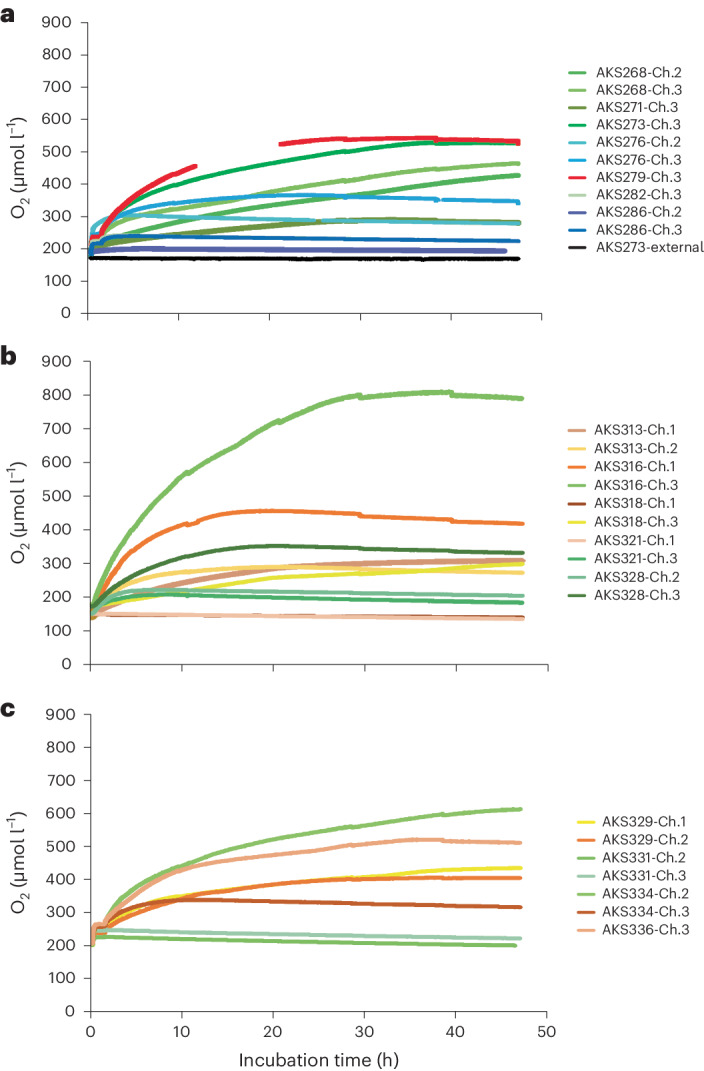
Oxygen concentrations in μmol l^−1^ measured by calibrated O_2_ optodes through time in h in the different benthic chamber incubations. **a**–**c**, The in situ benthic chamber lander deployments were made during the 5D (**a**), 5E (**b**) and 7A (**c**) cruises to the NORI-D license area (Extended Data Fig. 1). Nodules were present in all incubation experiments. The green hue, blue hue and red lines in the 5D figure (**a**) denote dead-algal biomass, dissolved inorganic carbon + NH_4_^+^ and filtered seawater treatments, respectively. The gap in the optode data in AKS279-Ch.3 was caused by the optode periodically not logging data. The black line indicates ambient O_2_ concentration measured on the outside of the benthic chambers during AKS273 on the 5D cruise. The green and yellow hue lines in the 5E (**b**) and 7A (**c**) figures denote the dead-algal biomass and control (no injection) treatments, respectively. The minor drops seen in some of the O_2_ concentration profiles at 28, 38 and 47 h are caused by the dilution of the chamber water with 50 ml of seawater that was entrained from the outside into the chamber through a 1.5 m (0.25 cm diameter) open tube when the syringe sampler collected seawater samples from within the chamber. The constant O_2_ concentration measured during the first 2 h of the 5D and 7A experiments was due to the stirrers being turned off for 1 h to allow the substrates (for example, dead-algal biomass) to sink to the sediment surface. Stirrers were turned on during the 5E expedition from the moment the lander was deployed until the lander returned and power to the stirrers was disconnected. Source data

**Extended Data Fig. 2:**
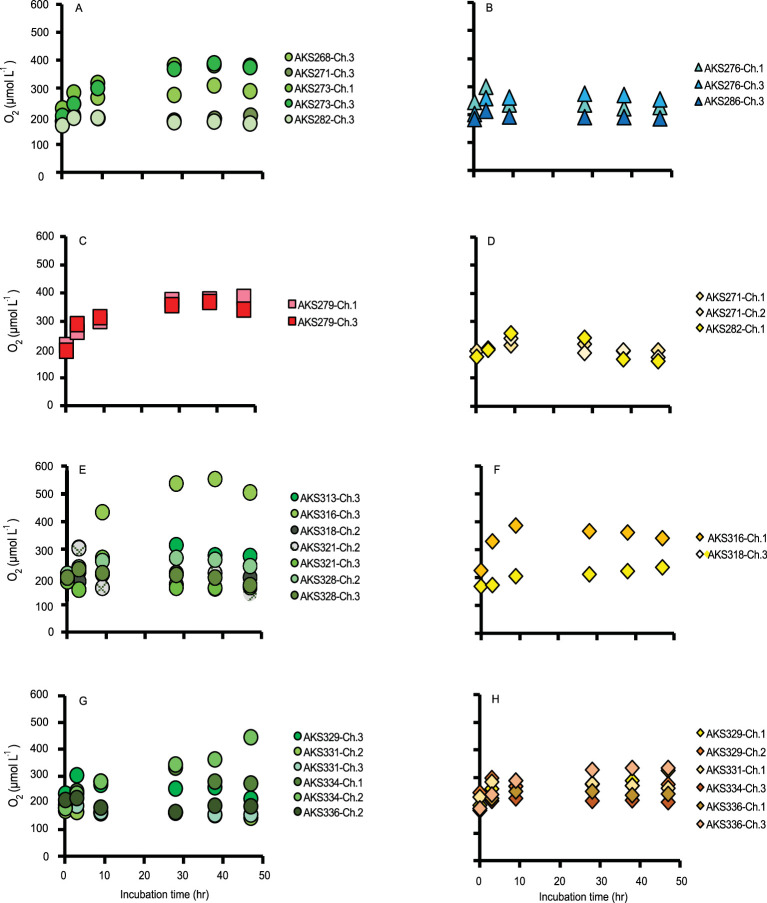
Oxygen concentrations measured from water samples by Winkler titration during the NORI-D benthic chamber lander experiments. Mean O_2_ concentration (μmol L^−1^) measured by micro-Winkler analysis conducted on water samples that were collected periodically from the chambers through time (hr) under different treatments. The treatments were dead-algal biomass during expeditions 5D (A), 5E (E), and 7A (G), DIC + NH_4_^+^ during expedition 5D (B), 0.45-μm filtered seawater during expedition 5D (C), and control (no injection) during expeditions 5D (D), 5E (F), and 7A (H). Each datapoint is the mean of two Winkler measurements. Source data

**Extended Data Fig. 3:**
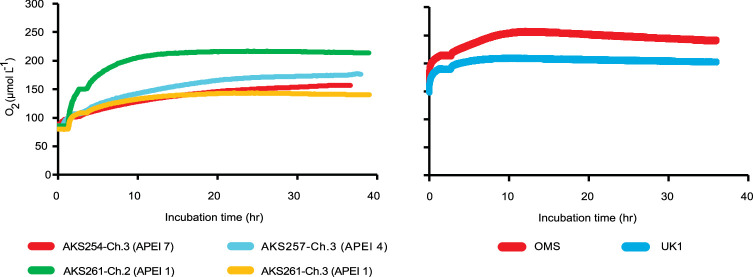
Oxygen optode concentrations measured during benthic chamber lander experiments in the UK1 and OMS license areas and APEIs 1, 4, and 7. Oxygen optode readings through time (hr) from 36-hour abyssal (4037-5216m) in-situ benthic chamber lander experiments conducted in the UK1 and OMS license areas in 2015 and APEIs 1, 4, and 7 in the western CCZ in June 2018. The experiments that were conducted were identical to those carried out at NORI-D. The O_2_ concentrations recorded by the optodes in the 2015 and 2018 experiments were derived from factory calibrations undertaken 4–6 months prior to the expeditions as in-situ temperature could not be replicated onboard during the optode calibration process. As such, only relative changes in O_2_ concentrations can be interpreted. Source data

Constant linear decreases in O_2_ optode readings were observed in two experiments (Fig. 1), and SCOC determined by in situ O_2_ microprofiling was 0.7 mmol O_2_ m^−2^ d^−1^ indicating that SCOC occurs in NORI-D as in many abyssal habitats^2–4^. However, O_2_ concentrations in 25 benthic chamber incubations started at 185.2 ± 2.9 µmol l^−1^ (1 standard error (SE)) and reached O_2_ maxima between 201 and 819 µmol l^−1^ over 47 h (Fig. 1), indicating net dark O_2_ production (DOP) corresponding to rates of 1.7–18 mmol O_2_ m^−2^ d^−1^. Independent measurements of O_2_ concentration using the Winkler method also showed DOP (Extended Data Fig. 2), providing evidence that the optodes were not malfunctioning. No statistically significant difference in the total net O_2_ produced (maximum [O_2_] – initial [O_2_]; Extended Data Table 2) was found between chambers (ANOVA, *F*_2,9_ = 0.107, *p* = 0.900) or experimental treatments (ANOVA, *F*_3,9_ = 0.876, *p* = 0.489), ruling out any experimental bias. We found no difference in the total net O_2_ produced between cruises (ANOVA, *F*_2,12_ = 0.391, *p* = 0.684), though DOP was correlated to the average surface area of the nodules (Spearman’s correlation, *⍴* = 0.664, *p* = 0.031). A re-evaluation of in situ O_2_ optode data collected from 36-h benthic chamber experiments in the abyssal eastern and western CCZ (Extended Data Figs. 1 and 3) also showed DOP, indicating its occurrence in multiple locations across the CCZ. Our findings contrast with all published deep-sea benthic O_2_ flux studies and suggest that DOP may provide O_2_ for benthic respiration. Whereas the DOP measured was greater than SCOC, we would urge caution when temporally upscaling our results, as the nonlinear production of O_2_ suggests that DOP may not be continuous in nature. Moreover, the variance in DOP activity seen between experiments and its relationship to nodule surface area suggests DOP activity may change with nodule spatial density and type (for example, diagenetic versus hydrogenetic), so upscaling our results by area is also imprudent without additional studies.

**Extended Data Fig. 4:**
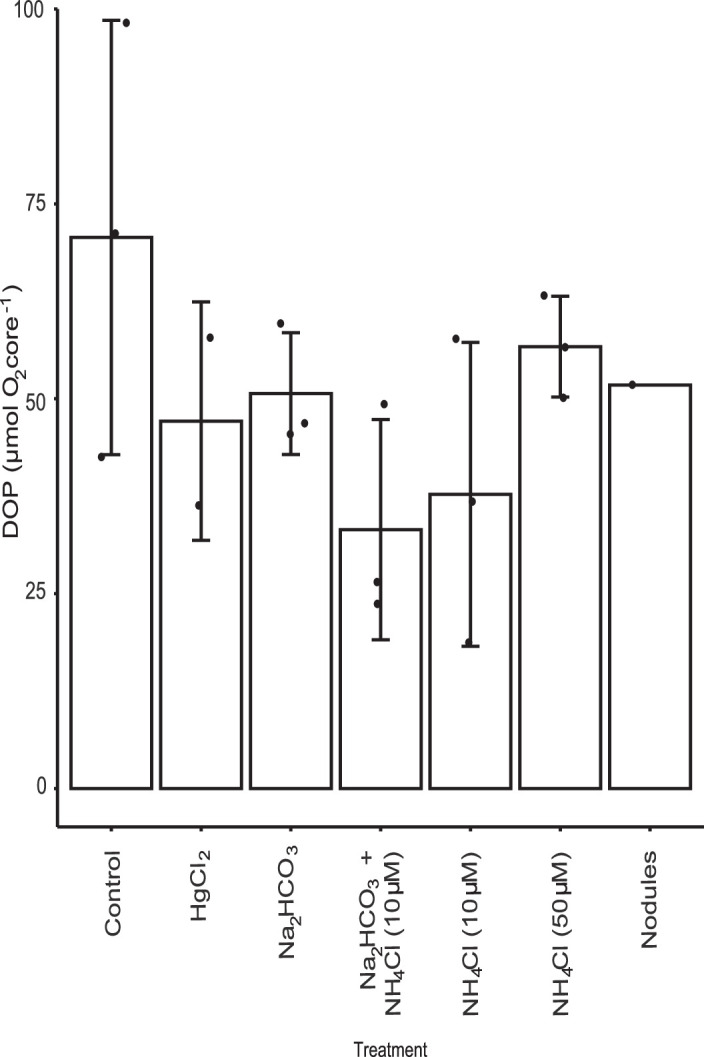
Bar chart showing total net O_2_ production in ex situ sediment cores. Mean total net O_2_ production (μmol O_2_ core^−1^) measured on sediment cores (n=1-3) exposed to a variety of treatments during 48-hr ex situ incubations that were carried out on the ship at in-situ temperature and in the dark during the 5D cruise. Oxygen production was determined from the difference in O_2_ concentration of the water phase overlying the sediment between t = 0 hours and 48 hours accounting for the core volume. Error bars refer to ± 1 standard deviation. Individual fluxes from the ex-situ incubations are also shown as data points overlying the bars. Source data

Several lines of evidence indicate that the DOP was not caused by experimental artefacts. First, the total O_2_ change between the experimental and control (non-injection) treatments was statistically indistinguishable, and a steady increase in O_2_ concentration was recorded over many hours in multiple experiments; these observations demonstrate that DOP was not attributable to the injection of exogenous fluids. Second, diffusion of O_2_ from trapped air bubbles within the chamber was unlikely because each chamber uses two one-way valves in the lid to purge air from the chambers as the lander sinks. Even if an air bubble could be trapped long enough to reach the seafloor, gaseous diffusion of O_2_ into the water phase would take < 1 s at 4,000 m depth (Extended Data Table 3), which is inconsistent with the steady increase in O_2_ over many hours seen in multiple experiments (Fig. 1). Third, intrusion of O_2_ from the plastic chambers into the water phase is unlikely (Methods) as they are built from polyoxymethylene, which is both highly inert and chemically stable in well-oxygenated settings and would not explain the variation in DOP because all experiments used identical materials. Last, DOP was also observed during 48-h ex situ sediment incubations (Extended Data Fig. 4).

**Extended Data Fig. 5:**
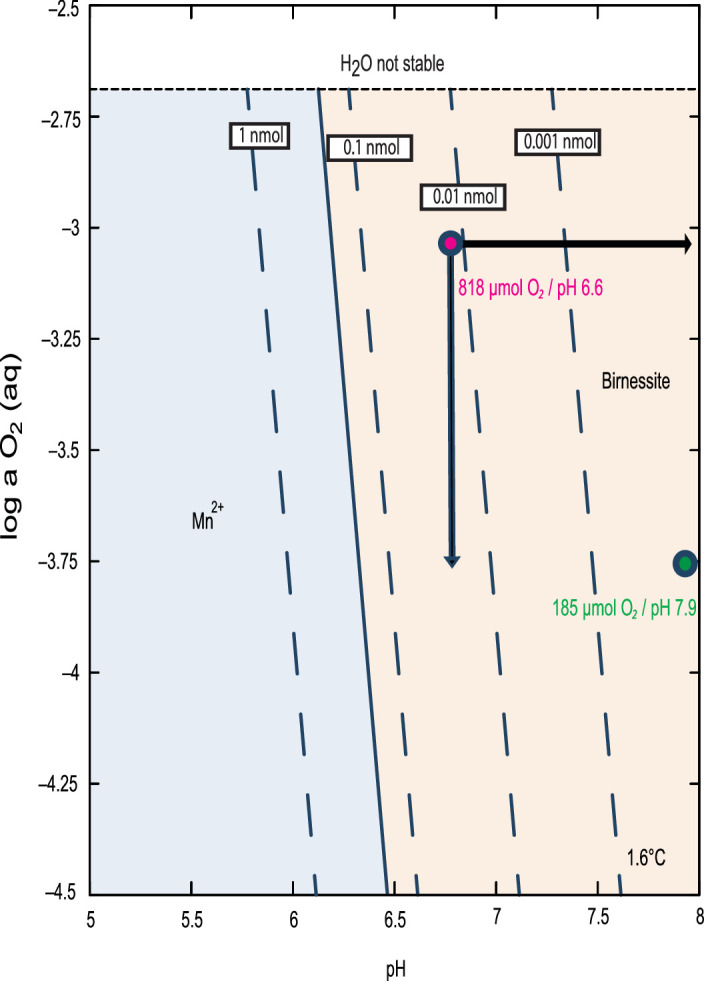
Phase stability and solubility of birnessite in seawater as a function of O_2_ activity and pH. The phase stability and solubility of birnessite (manganese [IV] oxide) in seawater as a function of O_2_ activity and pH at a temperature of 1.6 °C, 0.55M Cl, and 2e^−10^M Mn. The bold black line illustrates the phase boundary between birnessite and dissolved Mn^2+^; the dashed lines the solubility of birnessite into seawater. The green point indicates the predominant manganese form that would be experienced at the highest pH that was measured in MUC cores, and the lowest O_2_ condition (average bottom seawater); the red point indicates the predominant manganese form at the lowest pH (measured in MUC cores) and highest O_2_ concentration measured in the in-situ benthic chamber experiments at NORI-D with the arrows showing their range. Under the latter conditions, a vanishing small amount of birnessite would dissolve into seawater to form Mn^2+^.

Several lines of enquiry were pursued to explain the DOP. Subsurface advection of oxic bottom water from seamount flanks into seafloor sediments^5,6^ and then into the chambers was discounted based on in situ O_2_ microprofiling that showed pore water was a net sink for O_2_ and undersaturated compared with the O_2_ seen in the chambers. Furthermore, DOP was measured in sealed ex situ experiments (Extended Data Fig. 4) that prevented O_2_ intrusion from below. It is unlikely that biological mechanisms were responsible for the bulk of the DOP as ex situ core incubations revealed DOP in the presence of poison (HgCl_2_; Extended Data Fig. 4). Whereas many microbes in the CCZ are able to detoxify Hg (II) to Hg (0)^7^, and some microhabitat pore spaces in the core may have remained HgCl_2_ free, the taxa known to be capable of DOP (for example, *Nitrosopumilus maritimus*) are killed by its addition^8^. We also observed weak statistical support between the relative abundance of certain nitrogen-cycling microbial taxa and DOP (for example, Candidatus *Nitrosopumilus* *⍴* = 0.474, *p* = 0.420). The fact that DOP was detected in ex situ controls containing only polymetallic nodules (Extended Data Fig. 4) suggested that the DOP was linked to their presence. Hence, we estimated the potential contribution of radiolytic O_2_ production using a kinetic model^9^ and found 0.18 μmol l^−1^ O_2_ would be generated by this process within 48 h. We also modelled the chemical reduction of manganese (IV) oxide at in situ temperature (1.6 °C) across a range of pH and O_2_ conditions encountered at the seafloor to assess if this reaction (2MnO_2_ → 2MnO + O_2_;Extended Data Fig. 5) could liberate the O_2_ but found that <0.1 nmol of manganese (IV) oxide would be chemically reduced to manganese (II) at seafloor conditions. As such, localized radiolytic O_2_ production from the sediments and nodules and chemical dissolution explain only a negligible proportion (< 0.5%) of the DOP observed.

**Fig. 2:**
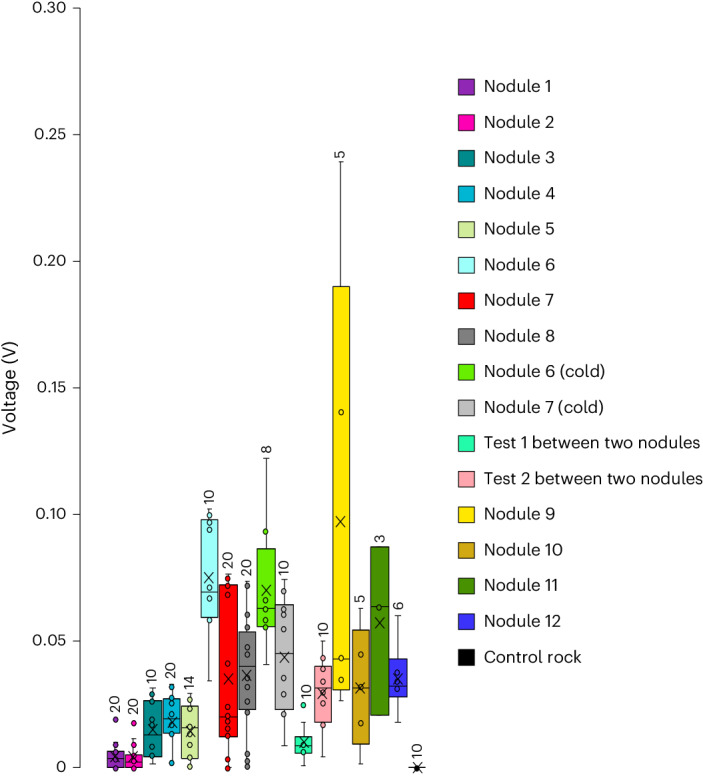
Box and whisker plots of background-corrected voltage potentials on nodule surfaces. The nodules were collected from the NORI-D (1-5), UK1 (6-8) and the BGR (9-12) license areas. Potentials were measured at 21 °C (nodules 1–12) and 5 °C (nodules 6 and 7 cold) and between two different UK1 nodules (Tests 1 and 2) and across the surface of a metamorphosed carbonate rock (control). Means are designated by the ‘x’ symbol, medians by the line, boxes show the lower and upper quartile values (excluding the median), whereas the whisker bars refer to the minimum and maximum data values. The number of technical replicate measurements made at different points on the surface of each nodule/rock to make each box-whisker is shown by the number above each whisker bar. Source data

The oxygen evolution reaction requires an input voltage of 1.23 V plus an overpotential of approximately 0.37 V to split seawater into H_2_ and O_2_ (ref. ^10^) at NORI-D’s seafloor mean pH (7.41). This value can be lowered by several hundred millivolts if the reaction proceeds via the lattice-oxygen-mediated mechanism^11^. Use of metal catalysts such as Mn oxides enriched with transition metals (for example, Ni) found in nodules^12^ and characterized by large tunnel areas and abundant defect sites can optimize the adsorption of reactants and enhance conductivity and catalytic performance^11,13,14^. We tested the electrical potential between two platinum electrodes at 153 sites on the surfaces of 12 nodules (Fig. 2) from the UK1, NORI-D and Bundesanstalt für Geowissenschaften und Rohstoffe (BGR) license areas. Although the potentials between different positions on the nodules were highly variable, - potentials up to 0.95 V were found and high mean background-corrected potentials were detected under cold-water conditions (Fig. 2 and Extended Data Table 4). On the basis of these studies and DOP being observed in nodule-only ex situ incubations (Extended Data Fig. 4), we hypothesize that the DOP may have partly resulted from seawater electrolysis, with the necessary energy coming from the potential difference between metal ions within the nodule layers, leading to an internal redistribution of electrons. Whereas questions remain concerning this potential mechanism (such as the identity of the energy source(s), longevity of DOP, catalytic stabilities, electrochemical conditions on exposed versus buried nodules surfaces and the influence of different chemistries within the nodule layers), the ‘geo-battery’ hypothesis was supported by the link between DOP and nodule average surface area. This connection could be due to an increased abundance of anode and cathode sites or a greater abundance of high Ni and Cu dendritic porous layers in larger nodules^15^. Assuming the ‘geo-battery’ is partly responsible for the DOP observed, the initial high DOP rate may have been related to the ‘bow-wave’ of the lander removing sediments from the surface of the nodules and exposing electrochemically active sites on the nodules. The slowdown in DOP seen later in the incubations could have then been caused by a reduction in voltage potential and/or degradation of metal-oxide catalysts that has been observed in Mn oxide catalysts previously^10^. Whereas this process requires further investigation, if true, DOP activity may fluctuate with sediment coverage on the nodules inviting the urgent question of how sediment remobilization and distribution over large areas during deep-sea mining may influence DOP.

Understanding the mechanism(s) behind DOP, its temporal nature and its spatial distribution will allow its role in abyssal ocean ecosystems to be better understood. Future studies of DOP in the deep sea may also shed light on broader relationships between metal-oxide deposition, biological evolution and the oxygenation of Earth^16,17^.

## Methods

A benthic chamber lander was deployed in the NORI-D license area six times in May–June 2021 (5D cruise), five times in November–December 2021 (5E cruise) and five times in August–September 2022 (7A cruise) (Extended Data Fig. 1 and Extended Data Table 1). The lander comprised three independent, autonomous, square benthic chambers (484 cm^2^) separated by approximately <0.5 m. After arriving at the seafloor, the lander waited for 0.07–1.34 d before the chambers were pushed into the sediment to create an enclosed microcosm of the seafloor. Ten minutes into the incubation period, the enclosed chambers were injected with 50 ml of one of three solutions: (1) 0.45-µm-filtered, cold surface seawater containing 79.2 mg of freeze-dried *Phaeodactylum tricornutum* algae, (2) 32 µM Na_2_HCO_3_ and 40 µM NH_4_Cl dissolved in cold artificial seawater (salinity 35) and (3) 0.45-µm-filtered, cold surface seawater. On some occasions, the injection mechanism failed allowing the response to control (no injection) conditions to be measured. The seafloor in the study area had a temperature of 1.6 °C ± 0.006 °C (SE, *n* = 28) and a pH of 7.41 ± 0.05 (SE, *n* = 17). Immediately after the injection, the overlying water was mixed with a submersible stirrer at 60 rpm for 1 min before the stirrer was turned off that allowed any particulate substrates to settle for 1 h. After 1 h, the stirrer was then turned on again for the remainder of the experiments. During the 5E expedition, the stirrers were programmed to continually stir the overlying water even immediately after injection.

The syringe samplers removed approximately 50 ml of seawater from the water phase of each chamber at 0.1 or 0.03, 1, 3, 9, 28, 38 and 47 h into the incubation experiment. Oxygen optodes (CONTROS HydroFlash O_2_ manufactured by Kongsberg Maritime Contros GmbH) mounted in the lid of each chamber logged O_2_ concentrations in the chamber every 10 seconds throughout each experiment. Two days before the first lander deployment of each cruise, the optodes underwent a two-point, multi-temperature calibration using 0 and 100% O_2_ calibration solutions at 1.2, 7, 18 and 30 °C following the recommendations of Bittig et al. (ref. ^18^). On the 5D cruise, we also calibrated the sensors 2 d after the last lander experiment so we could estimate optode drift, which was negligible (0.27 µmol l^−1^ d^−^^1^) over the course of the six-week cruise. The 0% and 100% O_2_ saturation solutions were created by bubbling 0.45-µm-filtered surface seawater in a bottle sitting in a water-chilling/heating unit with N_2_ gas (0%) or an aquarium air bubbling unit (100%) for 30 min. The O_2_ concentration of the calibration solutions was confirmed in triplicate by Winkler titration. After incubating seafloor sediments for 47 h, the lander chambers were closed by a shutter door at the base of the chambers, and the chambers were then pulled slowly out of the sediment, which took 1 h. The lander was then recalled from the seafloor. In eight instances, the lander programme did not finish and the doors did not shut, preventing the sampling of sediment and determination of the volume of the water phase in the chambers (Extended Data Table 2). Once the lander was back and secured on deck, the chambers were opened and the water above the sediment removed via syphoning into a bucket. The distance from the top of the sediment to the base of the chamber lid was then measured in four places to get an accurate water depth for water volume estimates. Whenever possible, a photograph was then taken of the chamber sediment and nodules from directly above the opening of the chamber. All syringes containing water samples were removed and taken to the shipboard lab for immediate processing or stored in a cold lab (4 °C) before processing. The optodes were removed and their onboard data downloaded to a computer. Finally, the nodules were removed from the chambers and washed of attached organic debris with cold (4 °C), 0.45-µm-filtered surface seawater and placed in sterile Whirlpak bags to be weighed in the laboratory later. The number of polymetallic nodules at the seafloor determined from chamber counts was 1170 ± 97 m^−2^.

Unfiltered syringe sample seawater was carefully transferred from each 50 ml syringe to a 12 ml exetainer via a 10 cm tube attached to the syringe nozzle, ensuring no air bubbles were introduced and immediately fixed for microWinkler titration. The sample was then mixed thoroughly using a glass bead placed in the exetainer and placed in the dark in a 4 °C refrigerator for 30–45 min to allow the precipitate to settle. Once the precipitate had sedimented, the exetainers were shaken again and left for 2–3 h before Winkler titrations were performed. All titrations were completed within 12 h after sampling to determine dissolved O_2_ concentrations. Each Winkler sample (approximately 5 ml) was titrated twice, and duplicate measurements showed minor differences in O_2_ concentration (5D cruise error: 3.5 ± 0.3 μmol l^−1^, *n* = 71; 5E cruise error: 1.3 ± 0.2 μmol l^−^^1^, *n* = 69; 7A cruise error: 2.8 ± 0.4 μmol l^−^^1^, *n* = 84). Winkler O_2_ concentration data were averaged for each syringe sample. The O_2_ concentrations estimated by Winkler analysis were 22 ± 1% (*n* = 42, SE, 5D cruise), 8 ± 4% (*n* = 39, SE, 5E cruise) and 24 ± 2% (*n* = 40, SE, 7A cruise) lower than the concentrations measured by the optodes at the same time point in the same incubations most likely due to out gassing of supersaturated O_2_ caused by depressurization and warming of the externally mounted syringes (whose samples were used for Winkler analyses) during the lander recovery to the surface.

Back on shore, the final O_2_ concentration values were calculated following Bittig et al. (ref. ^18^) from the optode, calibration and in situ pressure data that was derived from the depth where each lander deployment was made. Time stamps in the optode data were compared to the lander computer programme times so the optode readings could be aligned to the schedule of the chamber experiment. The total change in O_2_ concentration in each chamber was then calculated from the volume of the water phase above the sediment and the difference in O_2_ concentration from when the chambers started to seal off the sediment to the point when the maximum O_2_ concentration was reached.

### Benthic O_2_ microprofiling

Benthic O_2_ microprofiles were made during lander deployments AKS313, AKS316, AKS318 and AKS321 during the 5E cruise using a UNISENSE deep-sea microprofiling unit mounted <0.5 m from the benthic chambers. The microprofiles were made using 20 cm O_2_ microsensors that penetrated the sediment in 0.05 mm steps. The microsensors were calibrated 2 h before the lander deployments at in situ temperature (1.6 °C) at 0% and 100% O_2_ saturation (above). At each sampling depth, the microsensor stopped for 5 s before each measurement was made. The sensor then recorded five individual O_2_ concentration measurements. The average of these five measurements was taken for each depth point. The sediment surface was determined manually based on the turning point in the slope of O_2_ concentration with depth where O_2_ started to become depleted. SCOC was determined from Fick’s first law of diffusion.

### Microbiology sampling

Nodule and sediment samples for microbial community analyses were collected from the 5D experimental chambers. Approximately 30 g of sediment from each of the 0–2 cm and 2–5 cm horizons and 50 g of intact nodules were placed in separate sterile Whirlpak bags with a pre-sterilized spatula and then transferred to a −80 °C freezer. DNA from approximately 10 g of nodules and 250 mg of sediment were extracted using the Qiagen PowerMax soil and PowerSoil extraction kits, respectively. Extracted DNA was then shipped on dry ice to Laragen Inc. and sequenced using a proprietary in-house method. The V4 region of 16 S rRNA genes were amplified using the Earth Microbiome Project protocol^19^ with the 515 F (5′‐GTGYCAGCMGCCGCGGTAA^20^) and 806 R (5′-GGACTACNVGGGTWTCTAAT^21^) primers. Raw fastq files were processed using a custom pipeline (https://github.com/Boston-University-Microbiome-Initiative/BU16s) built with QIIME 2020.2 (https://www.nature.com/articles/s41587-019-0209-9). Adaptor sequences were removed using cutadapt (10.14806/ej.17.1.200), read truncation positions were determined by mineer (more below), amplicon sequence variants (ASVs) were generated using dada2 (trunc-len-r^20^) (10.1038/nmeth.3869) and ASVs were clustered to 99% identity with the SILVA 132 database (https://academic.oup.com/nar/article/42/D1/D643/1061236) using the vsearch cluster-features-closed-reference (10.7717/peerj.2584). Due to drops in sequencing quality, all reverse reads were truncated by 49 bases (from a length of 301 to 252) as determined by minERR, an algorithm for determining optimal sequence length based on sequence quality scores (https://github.com/michaelsilverstein/mineer). Family- and genus-level abundance was computed by summing the relative abundance of all ASVs with the same family/genus classification within each sample. Spearman correlations were then computed between family- and genus-level abundance and observed optode-derived total O_2_ changes. Sequences have been archived at National Centre for Biotechnology Information GenBank under the Bioproject ID PRJNA1117483.

### Polymetallic nodule surface area measurements

Photographs of the surface sediment and nodules in the chambers were imported into Image J. The outline of each nodule in each chamber photograph was then traced and the surface area of the nodule automatically calculated in Image J (assuming each surface nodule was flat in shape) and logged as an Image J file before being exported and saved as an Excel file.

### Radiolysis O_2_ production estimates

To estimate the potential radiolytic O_2_ production, published concentrations of ^238^U, ^235^U, ^232^Th, ^40^K (refs. ^22–26^) in seawater were used (Supplementary Table 1). For nodules, ^238^U, ^235^U and ^232^Th isotopes of three nodules from chamber experiments from the 5D cruise were measured by Multicollector-Inductively Coupled Plasma Mass Spectrometer using previously described methods^27–29^ and averaged; ^40^K values were derived from the literature^12^. Nodule and seawater contributions were calculated using a kinetic model developed by ref. ^9^ that incorporates 32 reactions (equation (1) in ref. ^30^). The nodule boundary layer was assumed to be fully integrated with the seawater, surpassing the respective ~23 to ~452 μm stopping power distance of alpha and beta particles used to model geologic materials^31^. Sediment radiolytic O_2_ was calculated as half of the previously quantified H_2_ production rates in equatorial Pacific subsurface sediment^32^, given the stoichiometry of water’s radiolytic decomposition (an equivalency that probably offers an overestimate of derived O_2_). Contributions from these three components (nodules, sediment and seawater) were scaled by the benthic chamber’s size and contents to produce an estimate of 0.18 μmol l^−1^ of O_2_ generated over 48 h according to the following expression.

$${({{\rm{O}}}_{2})}_{{{t}}}=({{{Q}}}_{{\mathrm{iz}}}\times {{{E}}}_{{\rm{a}}}\times{{G}}({{\rm{O}}}_{2})\times {{{M}}}_{{\rm{O}}2}\times{{{{A}}}_{{\mathrm{iz}}}}^{-1}\times {10}^{-2})\times (1-{{\rm{e}}}^{-\lambda {{t}}})$$

Here (O_2_)_*t*_ is the mass (kg) of O_2_ produced over a given time *t* (yr), *Q*_iz_ is the mass (g) of the isotope, *E*_a_ is the average energy (eV) released from the decay of one atom; *G*(O_2_) is the radiation chemical yield of molecules per 100 eV of the radiation energy; *M*_O2_ is the O_2_ molecular mass (g), *A*_iz_ is the isotope atomic mass (g) and *λ* is the isotope-specific decay constant (y^−1^). The overall (O_2_)_*t*_ value summed the contributions from ^238^U, ^235^U, ^232^Th and ^40^K across water, nodule and sediment sources.

### Electrochemistry measurements

Voltage potentials were measured using a Keithley DMM6500 digital multimeter on nodules previously collected by coring in the UK1, NORI-D and BGR license areas. Nodules were initially immersed for seven days in Instant Ocean artificial seawater (salinity 35). To measure the potentials, two electrodes (platinum wire, 99.9% purity) were first washed in perchloric acid, rinsed in Milli-Q water and dried before being attached to alligator clamps attached to the multimeter. The platinum wires were then immersed in Instant Ocean artificial seawater in a glass petri dish to measure background voltages (0.003 ± 0.001 V, SE, *n* = 17) until stable. Once stable, a nodule was placed in the petri dish and the platinum probes placed on the nodule at random locations, ensuring contact in one of two ways. We either carefully drilled a hole into some nodules so one platinum wire could be fixed inside it while the second platinum wire was firmly pressed against the nodule surface using a clamp. Alternatively, the platinum wires were pressed firmly against two different spots on the nodule surface and held in place using a clamp. Voltages were then recorded for 1–2 min until the signal was stable. This procedure was repeated up to 20 times in different randomly selected regions of the nodules depending on their size. Measurements were undertaken on 12 nodules at 21 °C (*n* = 153) and a single control rock composed of metamorphosed carbonate (*n* = 10). Two nodules from UK1 were also retested after being cooled to 5 °C (*n* = 18) by placing them in Instant Ocean water in a refrigerator overnight. Voltage potentials (*n* = 20) between two nodules were measured using four nodules collected from UK1. Potentials measured during each measurement were averaged and corrected for the background seawater voltage measured using only Instant Ocean seawater in the absence of a nodule. Measured resistances inside some of the nodules that were broken up were in the kΩ to 100s of kΩ range, though it is unclear if these resistivities change at the nano- or microscale requiring further investigation.

### Geochemistry modelling

The chemical stability and solubility of manganese (IV) oxide (birnessite) to dissolved Mn^2+^ as a function of pH and O_2_ activity was modelled using the Geochemist Workbench Professional (version 12) software, with the in-built and internally consistent THERMO database. The conditions used for generating the phase diagram (Extended Data Fig. 5) represent bottom seawater as measured in the eastern CCZ with a temperature of 1.6 °C and chlorine and manganese concentrations of 0.55 M Cl and 2e^−10 ^M Mn, respectively.

### Ex situ core incubations

Opportunistic ex situ experiments were undertaken during the 5D cruise using sediment cores retrieved by a multi-corer from the CTA area (Extended Data Fig. 1). Immediately after the multi-corer arrived back at the surface, cores were removed and transferred to a cold lab held at in situ temperature. The cores were then exposed to the following five treatments (administered using a 60 ml syringe), which included (1) Na_2_HCO_3_ (0.3 μM final concentration, *n* = 3), (2) NH_4_Cl (10 μM final concentration, *n* = 3) and (3) NH_4_Cl (50 μM final concentration, *n* = 3), (4) 0.3 μM Na_2_HCO_3_ + 10 μM NH_4_Cl (final concentration, *n* = 3) and (5) HgCl_2_ (1.1 μM final concentration, *n* = 3). No-injection controls (*n* = 3) were also performed and separate core experiments in which four nodules were incubated for 48 h by themselves with no additions. After addition, the water phase of each core was stirred and a 50-ml sample of top water was taken for microWinkler analysis (as above). Stoppers were then placed on the top of the cores, ensuring no air bubbles were present. The stoppers were secured tightly and the cores fully submerged in a large bucket containing 0.45-µm-filtered, cold, surface seawater (salinity 35). The bucket was covered with five black plastic bags and secured in the cold room with the lights turned off. After 48 h, the cores were removed from the bucket, and the cores were inspected for the presence of air bubbles. Only one core, a HgCl_2_ treatment, had a gas bubble beneath the bung, which was rejected from further analysis, leaving *n* = 2 for this treatment. The other cores were then re-sampled for dissolved O_2_ and analysed as before. Core-specific water volume measurements were used together with the change in O_2_ concentration to calculate the total net O_2_ change per core.

To determine if our ex situ DOP detection was affected by intrusion of O_2_ from the atmosphere into the core tube, two controls were performed: a shipboard test with an O_2_ microprofiler and a lab-based test using the Winkler method. Shipboard, a clean core tube was filled with Milli-Q water and sparged with N_2_ for 10 min before beginning the test. A Metrohm 8663 Multimeter was inserted through a predrilled hole in the rubber stopper, allowing for O_2_ concentration to be recorded every 5 s. An increase from 39 to 69 µmol l^−1^ was observed over ~5 h, corresponding to a rate of 0.14 mmol m^−2^ d^−1^ or 4% of the 3.5 mmol m^−2^ d^−1^ mean net DOP measured in the ex situ experiments. Back in the home laboratory, three of the original core tubes were filled with 4 °C, 0.2-µm-filtered artificial seawater (salinity 35) and sparged with N_2_ for 8 min through a filtered pipette tip to achieve an initial dissolved O_2_ concentration of ~100 µmol l^−1^ (for example, the approximate starting O_2_ concentrations for the shipboard experiments). The tubes were sealed with rubber stoppers and electrical tape, being careful to avoid bubble formation. They were then submerged in a 32-gallon plastic garbage can of unfiltered seawater (O_2_ concentration: 228.12 µmol l^−1^) in a dark cold room (8 °C) for 48 h. After 48 h, the tubes were quickly unsealed and analysed one at a time to prevent additional O_2_ dissolution from the air. A 50-ml sterile syringe was used to slowly collect 10 ml of seawater from the centre of the core tube, being sure to avoid bubble entrainment into the syringe. The sample was carefully expelled into a 10-ml reaction vial and fixed using the adjusted values for a 10-ml sample according to a volume-scaled Winkler titration protocol^33^ and the reagents from the LaMotte Dissolved Oxygen Test Kit. The fixation of each collected sample was done in less than 2 min in a fume hood. Dissolved O_2_ increased by 0.11 mmol m^−2^ d^−1^ during the 48 h, which corresponds to between 3.2% of the mean net DOP rate observed in the ex situ experiments (3.5 mmol m^−2^ d^−1^). Both of our control experiments provide high confidence that the diffusion of external O_2_ into the core tubes did not cause the O_2_ production measured in the ex situ core incubations.

### Calculations to quantify intrusion of O_2_ from the polyoxymethylene chambers and lids

Oxygen intrusion was estimated from Stephens^34^ who calculated that 20.66 µmol l^−1^ of O_2_ could diffuse out of 428 cm^2^ of polyoxymethylene plastic when immersed for 48 h in hypoxic water (O_2_ diffusion rate: 0.02 µmol O_2_ cm^−2^ d^−1^). To determine the total area of plastic that would be available for diffusion (869–1,584 cm^2^), we added the surface area of the lid to the surface area of the four walls that would be exposed at the seafloor (based on the depth of the water phase—above). The minimum and maximum areas available for diffusion were multiplied by 0.02 µmol O_2_ cm^−2^ d^−1^ to estimate that 41.9–76.5 µmol O_2_ l^−1^ would diffuse out of the polyoxymethylene chamber walls and lid in 48 h under hypoxic conditions. Thus, we are highly confident that O_2_ leakage from the plastic chambers could not replicate the high O_2_ concentration seen in some of our oxygenated experiments (Fig. 1).

## Online content

Any methods, additional references, Nature Portfolio reporting summaries, source data, extended data, supplementary information, acknowledgements, peer review information; details of author contributions and competing interests; and statements of data and code availability are available at 10.1038/s41561-024-01480-8.

## Supplementary information

Supplementary Information Supplementary Table 1 and caption.

## Source data

Source Data Fig. 1 Oxygen optode concentration (μmol l^−1^) data from benthic chamber lander experiments made during cruises 5D, 5E and 7A.

Source Data Fig. 2 Voltages (V) measured on the surface of nodules from the NORI-D, UK1 and BGR license areas.

Source Data Extended Data Fig. 2 Oxygen concentrations (μmol l^−1^) determined by Winkler titration on syringe samples collected from benthic chamber lander experiments made during cruises 5D, 5E and 7A.

Source Data Extended Data Fig. 3 Oxygen optode concentration (μmol l^−1^) data from benthic chamber lander experiments made during research cruises to the OMS, UK1 and APEI 1, 4 and 7 areas.

Source Data Extended Data Fig. 4 Oxygen concentrations (μmol l^−1^) determined by Winkler titration from the ex situ experiments conducted during the 5D cruise.

## Figures and Tables

**Extended Data Table 1:**
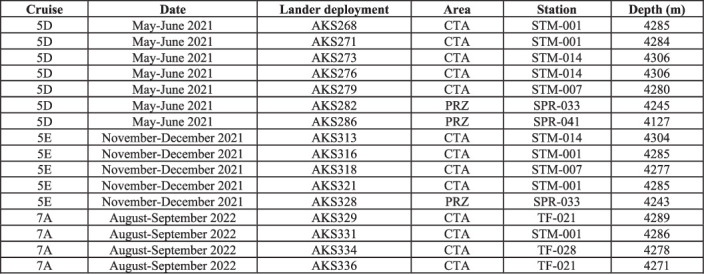
In-situ benthic chamber lander deployment locations and depths in NORI-D

In-situ benthic chamber lander deployment locations and depths from the 3 cruises to the NORI-D license area. Deployment areas are shown in Extended Data Fig. 1 and are defined as the Collector Test Area (CTA) and Preservation Reference Zone (PRZ).

**Extended Data Table 2:**
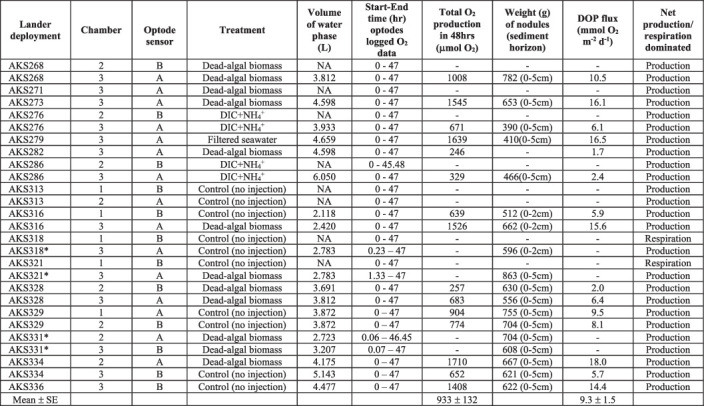
Total net oxygen change measured by O_2_ optodes during the in-situ benthic chamber lander experiments at NORI-D

Total net oxygen change measured by O_2_ optodes during the in-situ benthic chamber lander experiments at NORI-D. Stars (*) denote the incubations where the optodes did not start to log O_2_ concentrations immediately or stopped logging before 47 hr meaning the total O_2_ change could not be determined (designated by a dash [-]). The total net O_2_ change could also not be determined in a sub-set of experiments (designated by a dash [-]) because the chambers failed to seal sediments at the end of the experiment, which meant that the volume of water in the chamber could not be determined (designated NA) once the lander arrived back on deck. DOP flux rates were determined from the change in O_2_ concentration from the point when the optodes started logging to t = 47-hr. The determination of net production/ respiration dominated experiments was based on the O_2_ optode profiles seen in Fig. 1.

**Extended Data Table 3:**
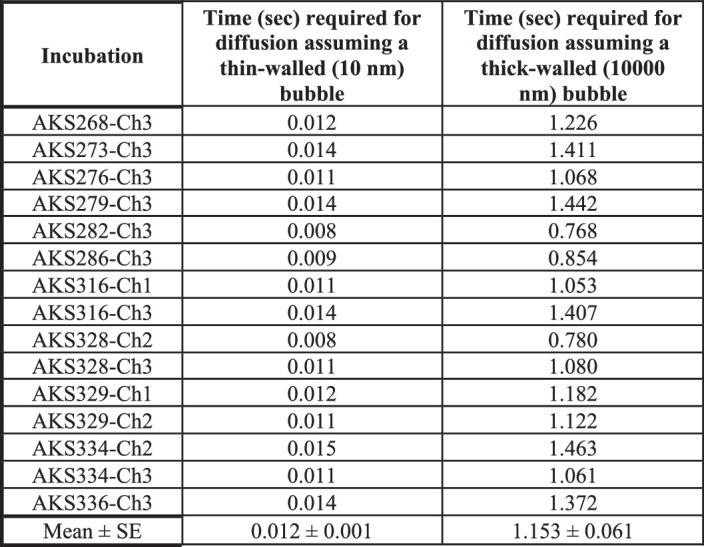
Theoretical diffusion times for thin versus thick-walled bubbles at the seafloor

Theoretical diffusion times (seconds) for thin versus thick-walled bubbles at the seafloor. The theoretical time for O_2_ to diffuse from a trapped air bubble at the seafloor was estimated by calculating the size of air bubble that would be required at the surface to produce the O_2_ increase seen in each chamber. This was estimated from the difference between the max O_2_ concentration value recorded by the optode and the initial O_2_ optode reading in the chamber, the volume (L) of the water phase in the chamber and assuming air was comprised of 21% O_2_. The size of the bubble at the seafloor was then computed using Boyle’s law and the in-situ pressure calculated from the deployment depth. The O_2_ concentration gradient (dC) between the bubble and the water phase of the chamber was determined from the concentration of O_2_ in the theoretical bubble and the initial concentration of O_2_ in the chamber. The diffusion distance (dZ) was set at 10 and 10,000 nm to calculate the diffusion times across a thin versus thick-walled bubble. The diffusion coefficient for O_2_ (1.064 x 10^−5^ cm^−2^ s^−1^) was calculated from the O_2_ diffusion coefficient based on an in-situ salinity of 35 and temperature of 1.6^o^C. Fick’s first law of diffusion was then used to calculate the diffusion time based on the diffusion coefficient, dZ and dC.

**Extended Data Table 4:**
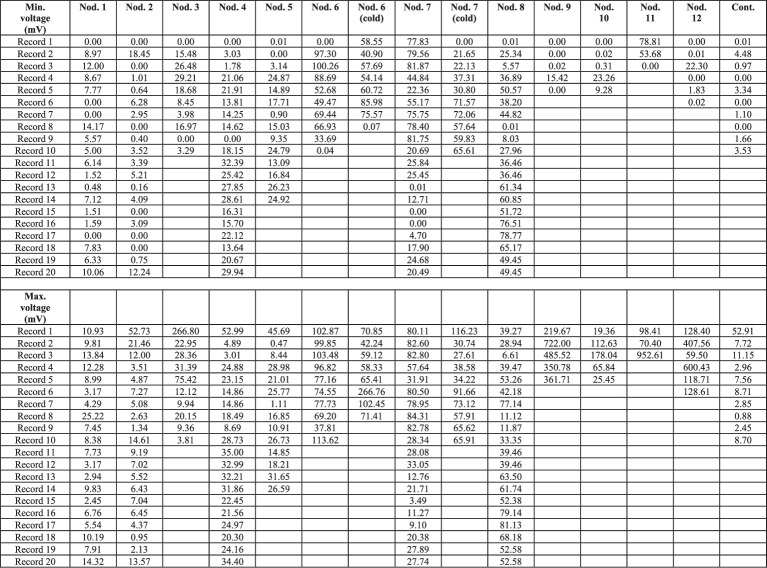
Minimum and maximum voltage potentials (mV) measured on the surface of polymetallic nodules

Minimum and maximum voltage potentials (mV) measured on the surface of the polymetallic nodules (nod.) at 21^o^C (nodules 1 – 12) and 5^o^C (nodules 6 and 7 cold), and a control (cont.) rock. The voltages have not been corrected for the background voltages measured using only instant water.

## Data Availability

Source data are provided with this paper. These data are also available via Dryad at 10.5061/dryad.tdz08kq6w (ref. ^35^), and geological samples were exported in accordance with relevant permits. The nucleotide sequences generated by metagenome sequencing have been deposited in the National Centre for Biotechnology Information database under BioProject ID PRJNA1117483.
